# Severe Hypercapnia Requiring 48-h Whole-Body Hypothermia in an Infant with Acute Bronchiolitis

**DOI:** 10.3390/children9091339

**Published:** 2022-09-01

**Authors:** Michela Librandi, Serena Scapaticci, Valentina Chiavaroli, Altea Petrucci, Paola Cicioni, Rita Cognigni, Francesco Chiarelli, Susanna Di Valerio

**Affiliations:** 1 Department of Pediatrics, University of Chieti—Pescara, 66100 Chieti, Italy; 2 Neonatal Intensive Care Unit, Pescara Public Hospital, 65123 Pescara, Italy; 3 Liggins Institute, University of Auckland, Auckland 1010, New Zealand

**Keywords:** hypothermia, hypercapnia, bronchiolitis, infant

## Abstract

Bronchiolitis is a clinical syndrome involving the lower respiratory tract of infants and young children. The majority of patients recover using adequate hydration and oxygen (O_2_) therapy, while a small number of patients require ventilatory assistance. Beyond these therapeutical approaches, there are no available strategies for patients that do not improve. Hypothermia is a measure used to prevent neonatal hypoxic–ischemic encephalopathy by preventing carbon dioxide (CO_2_) production and subsequent tissue damage. Other medical applications of hypothermia have been proposed, such as in acute respiratory failure and necrotizing colitis. **Case report:** We report the case of a 50-day-old girl hospitalized with severe bronchiolitis caused by respiratory syncytial virus. On admission, the girl presented severe hypercapnic respiratory failure, requiring intubation and ventilatory support with conventional and non-conventional systems. However, the patient’s general conditions worsened with elevated O_2_ demand, thus whole-body hypothermia was attempted and performed for 48 h, with a gradual improvement in the respiratory function. No adverse effects were detected. **Conclusions:** Whole-body hypothermia could have a critical role as a rescue treatment in infants affected by severe hypercapnic respiratory failure, at the expense of few and rare side effects (bradycardia, coagulopathy, hyperglycemia). Notably, beyond reducing CO_2_ production, whole-body hypothermia might have an impact in restoring lung function in newborns using bronchiolitis refractory to maximal medical therapy and invasive ventilation.

## 1. Introduction

Bronchiolitis is a common form of lower respiratory tract infection in infants less than 24 months old, most often of viral origin. Currently, the best therapeutic approach is supportive treatment based on adequate hydration and nutrition and, if necessary, oxygen (O_2_) therapy [1,2]. A low percentage of patients may require invasive or non-invasive ventilatory assistance.

Whole-body hypothermia is applied to newborns suffering asphyxia to improve their neurodevelopmental outcomes. Namely, the lowering of body temperature decreases O_2_ consumption and carbon dioxide (CO_2_) production, thus reducing cellular energy metabolism with protective effects on damaged tissues [3]. Based on these beneficial effects, hypothermic treatment has been applied to adult patients with acute respiratory distress syndrome [4,5]. In this regard, a recent case report has described the advantages of treating a patient affected with severe acute respiratory syndrome coronavirus 2, who developed severe acute respiratory distress syndrome resistant to mechanical ventilation, prone position, and muscle relaxants [6]. Mild hypothermia (temperature between 33.0 °C and 34.0 °C) has brought about rapid improvement in terms of hemodynamics, followed by gain in pulmonary function within 72 h. The possible use of hypothermia has been reported also in infants with others pathological conditions, such as acute respiratory failure and necrotizing colitis [4] Of importance, 24-h whole-body hypothermia has been performed as a rescue treatment for refractory hypercapnia in a 5-month-old infant with acute bronchiolitis [3].

This successful use of hypothermia has opened novel and effective therapeutic approach for intractable hypercapnia, which can be life threatening in infants suffering severe respiratory failure. We report the first case of severe hypercapnia requiring 48-h whole-body hypothermia in a female infant with acute bronchiolitis refractory to maximum ventilation assistance.

## 2. Case Presentation

In December 2017, a 50-day-old female Caucasian infant was admitted to the neonatal intensive care unit due to acute bronchiolitis with severe respiratory distress. Multiplex real-time reverse transcription polymerase chain reaction (RT-PCR) analysis on a nasal swab sample for the qualitative detection of RNA was positive for respiratory syncytial virus. Simultaneously, influenza A virus (flu A) and influenza B virus (flu B) infection were excluded with the same method. The girl was the firstborn of unrelated healthy parents who did not suffer from any respiratory illnesses and denied exposure to sick people. The infant was delivered by uneventful vaginal delivery at 37 weeks and three days. The birth weight was 2980 gr (0.29 SDS, 61st percentile). She did not present any health problems in the first days of life, and showed regular physical growth during the first weeks of life.

Two days before hospitalization, the infant presented with a cough, rhinitis, and inappetence. Upon later appearance of respiratory distress, she was conducted to the emergency department. On admission, the girl was afebrile. Physical examination demonstrated severely compromised general conditions, with wheezing, dyspnea, and decreased breathing sounds on auscultation of lungs. Blood tests showed a clinical picture of severe hypercapnic respiratory failure (on capillary blood sample: pH 7.25, PaCO_2_ 71.4 mmHg, HCO_3_ -31.3 mg/dL, excess base 3.9). Sepsis was excluded. A chest X-ray showed signs of severe respiratory distress. Echocardiography excluded congenital heart defects. The infant was immediately intubated, and conventional mechanical ventilation with synchronous intermittent positive pressure ventilation (SIPPV) was started, with constant monitoring of vital parameters. After placing a central venous line and a nasogastric tube, adequate sedation was provided and total parenteral nutrition was initiated. Simultaneously, inotropic agents (dopamine and dobutamine) were administered to offer support to the cardiovascular system; this strategy was not adopted with the support of targeted neonatal echocardiography (TnEcho), which was unavailable in the neonatal intensive care unit. Antibiotic prophylaxis with cephalosporine and macrolides (ceftriaxone and clarithromycin, respectively) was started. Despite the ongoing invasive ventilatory strategy, the patient’s general conditions worsened with increased O_2_ demand. After approximately 20 h of conventional mechanical ventilation, high-frequency oscillatory ventilation was started. Nevertheless, blood gas monitoring showed a progressive increase in CO_2_ level. 

On the third day of hospitalization, exogenous surfactant (poractant alfa; Curosurf, Chiesi Farmaceutici, Parma, Italy) was administered via intratracheal instillation, followed by a bronchoalveolar lavage with exogenous surfactant during the next day. Despite the ongoing medical and ventilator strategies, hypercapnia progressively worsened, reaching a capillary PaCO_2_ level of 123 mmHg. At this point, rescue treatment with whole-body hypothermia (34.0 °C) was started in an attempt to reduce CO_2_ production. Of note, the infant showed gradual respiratory improvement with a decrease in CO_2_ level (Table 1). To further enhance gas exchanges by increasing lung vasodilatation, nitric oxide by inhalation was started. Overall, whole-body hypothermia was maintained for 48 h, with further lowering of capillary PaCO_2_ level (Table 1) and no side effects. Afterwards, the baby was gradually warmed up by increasing the core temperature by 0.25 °C/h, without detecting a subsequent CO_2_ increase (Table 1). High-frequency oscillatory ventilation was maintained for five days, after which SIPPV was started. The need for O_2_ therapy progressively reduced. Overall, nitric oxide was provided for 10 days. Extubation was performed after 10 days of hospitalization, with a switch to non-invasive mechanical ventilation (high-flow nasal cannula) for two days. Hemodynamic support lasted six days, while antibiotic prophylaxis was discontinued on day nine. During hospitalization, no neurological deficits were observed. To exclude the injury to the central nervous system caused by severe hypercapnia, the infant underwent a brain ultrasound, electroencephalography, and magnetic resonance imaging, the results of which were normal. Overall, after 20 days spent in the hospital, the patient was discharged due to her good general condition. The infant was followed over the next few months without showing any growth or nervous/cognitive disorders.

## 3. Discussion

This clinical case supports and highlights the beneficial use of hypothermia to treat infants suffering from severe hypercapnic respiratory failure, which is refractory to maximal medical therapy and invasive ventilation. Whole-body hypothermia is applied to newborns suffering hypoxic ischemic encephalopathy to improve their neurodevelopmental outcomes [7]. This approach requires the achievement of a core body temperature of 33.5 °C maintained for 72 h [8]. Indeed, the lowering of body temperature decreases O_2_ consumption and the resultant CO_2_ production, thus reducing cellular energy metabolism with protective effects on damaged tissues.

Over the last decade, several studies reported encouraging results about alternative applications of hypothermic treatment in the medical field. Studies on preterm lambs [9] have suggested a positive effect of cooling during acute respiratory failure, during which there is gas exchange impairment leading to hypoxia and hypercapnia [10]. Furthermore, Aslami et al. reported an increase in oxygenation and ventilation during hypothermic treatment in adult mechanically ventilated patients after cardiac arrest [11]. Hypothermia acts by lowering cellular metabolic rate, leading to a reduction in O_2_ demand and CO_2_ production. Animal studies have estimated that CO_2_ level lowers by approximately 50% during whole-body hypothermia [12]. Among the possibility of a raised metabolic demand during rewarming, a recent study has shown that only heart rate increases, while oxygen requirement, fraction of inspired oxygen, blood lactate level, and mean blood pressure do not significantly change [3]. This effect is probably related to the lung function improvement. Furthermore, it has been reported that a possible reduction in inflammatory mediators in newborn lungs during hypothermia coincides with improvement in respiratory condition [13]. In addition to its well-known anti-inflammatory and metabolic properties, Autilio et al. demonstrated how hypothermia leads to changes in surfactant composition and absorption [8]. Similarly, Dassios et al. showed that respiratory muscle contractility properties were enhanced by decreased temperature in a study involving 31 mechanically ventilated full-term newborn infants treated with hypothermia for hypoxic–ischemic encephalopathy [14]. Hypothermia is also able to inhibit the inflammatory cascade [15].

During the coronavirus (COVID-19) pandemic, mild therapeutic hypothermia has presented itself as an inexpensive and easily applicable therapy to be used as an alternative to extra corporeal membrane oxygenation, the use of which has been limited due to the discrepancy between the high number of patients who do not respond to ventilatory assistance and the capacity of most health systems. The authors propose that the lowering of body temperature might enhance gas exchange, which is typically increased during the hyperinflammatory state characterizing COVID-19 patients [6]. Additionally, mild hypothermia reduces cardiac output without altering anaerobic oxygen consumption. It is not yet clear how it acts exactly. It is probable that the shift to the left of the dissociation curve of hemoglobin, the increased diffusion of oxygen to tissues, the regulation of the microcirculation shunt in end-organ parenchyma, and the improved oxygen utilization by tissues all promote improvement in the cardiovascular system [4,5].

The possible use of hypothermia has also been proposed in infants with acute respiratory failure. Namely, in 2012, whole-body hypothermia was performed for 24 h as a rescue treatment for refractory hypercapnia in a 5-month-old infant with acute bronchiolitis [12]. This successful use of hypothermia has opened a novel and effective therapeutic approach for intractable hypercapnia, which can be life threatening. No additional studies have been performed to clarify the potential beneficial effects of hypothermic treatment in infants with acute respiratory failure refractory to maximum ventilation. Of importance, our clinical report is the first case of severe hypercapnia in an infant affected by respiratory failure that was successfully treated with 48-h whole-body hypothermia. Unfortunately, in our intensive care setting, we were unable to use TnEcho as a non-invasive technique to evaluate cardiovascular performance and systemic hemodynamic. 

In conclusion, further studies are needed to evaluate and possibly confirm the beneficial effects of whole-body hypothermia in infants suffering from severe respiratory distress, such as in acute bronchiolitis with hypercapnia refractory to maximum ventilation assistance. Moreover, further information needs to be obtained to know when hypothermic treatment should begin and for how long it should be maintained.

## Figures and Tables

**Table 1:** Vital signs, blood gas values and ventilatory parameters of the infant before, during and after therapeutic whole-body hypothermia.

Parameters	Pre-Hypothermia	Hypothermia Day 1	Hypothermia Day 2	Rewarming	Normothermia
**Temperature (°C)**	36.5	34	34	35.5	36.5
**pH**	7.04	7.22	7.39	7.42	7.44
**Lactate (mmol/l)**	0.3	1	0.9	1.3	0.6
**PCO_2_ (mmHg)**	123	71.5	59.4	49.3	43.6
**Sp0_2_ (%)**	97	97	98	98	99
**Heart rate (bpm)**	148	143	123	131	130
**MAP**	14.5	20	17.5	15.5	13.5
**Fi02 (%)**	60	45	35	30	28

Legend: PC0_2_, partial pressure of carbon dioxide; Sp0_2_, arterial oxygen saturation; bpm, beats per minute; FiO_2_, fraction of inspired oxygen.

## Data Availability

Data sharing not applicable—no new data generated.
